# A 45-year-old Female with an Atypical Presentation of Pharyngitis

**DOI:** 10.5811/cpcem.2020.2.46974

**Published:** 2020-04-27

**Authors:** Artur Schander, Andrew A. Glickman, Nancy Weber, Brian Rodgers, Michael B. Carney

**Affiliations:** * Sacred Heart Hospital, Department of Emergency Medicine, Pensacola, Florida; † HCA/USF Morsani College of Medicine GME Consortium: Brandon Regional Hospital, Department of Emergency Medicine, Brandon, Florida; ‡ Texas Tech University Health Sciences Center, Department of Emergency Medicine, El Paso, Texas; § Paul L. Foster School of Medicine, Department of Emergency Medicine, El Paso, Texas; ** Dallas Ear Institute, Dallas, Texas; †† Reynold’s Memorial Hospital, Department of Emergency Medicine, Glen Dale, West Virginia

**Keywords:** HIV, AIDS, oral hairy leukoplakia, oral lesion

## Abstract

**Introduction:**

Emergency physicians are trained to treat a variety of ailments in the emergency department (ED), some of which are emergent, while others are not. A common complaint seen in the ED is a sore throat. While most sore throats are easily diagnosed and treated, less common causes are often not considered in the differential diagnoses. Therefore, the purpose of this case study was to present an atypical case of sore throat and discuss differential diagnoses.

**Case Presentation:**

The patient was a 45-year-old female who presented to the ED with a three-day history of sore throat that was exacerbated by eating and drinking. The patient was not on any prescription medications, but tried over-the-counter medications for the sore throat without any improvement in symptoms. Review of systems was positive for sore throat, fevers, and chills. Physical examination of her oropharynx revealed mildly dry mucous membranes with confluent plaques and white patchy ulcerative appearance involving the tongue, tonsils, hard palate, and soft palate. Rapid streptococcal antigen, mononucleosis spot test, and KOH test were performed and found to be negative.

**Discussion:**

After initial testing was negative, a follow-up complete blood count with differential and complete metabolic profile were ordered. The patient was found to have decreased lymphocytes and platelets. Based upon those results, a diagnosis was made in the ED, the patient was started on medication, and further laboratory workup was ordered to confirm the diagnosis. ED providers should consider non-infectious as well as infectious causes for a sore throat, as this might lead to a diagnosis of an underlying condition.

## CASE PRESENTATION

A 45-year-old African-American female presented to the emergency department (ED) of a rural, academic medical center with a three-day history of “sore throat.” The patient rated her pain as a 7/10 and described the pain as a “burning pain and a raw sensation,” which was exacerbated by eating and drinking. She stated that she had tried over-the-counter (OTC) ibuprofen, lozenges, and oral benzocaine throat spray without any improvement in her symptoms. The patient admitted to a past medical history of hypertension, gastroesophageal reflux disease (GERD), depression, and bipolar disorder. However, she denied taking any medications for her medical conditions. She admitted to a one-pack-per-day smoking history and occasional alcohol use, but denied illicit drug use, specifically intravenous drug use (IVDU). The patient informed the practitioners that she had recently moved to the area; therefore she did not have a primary care physician, hence coming to the ED.

A review of her systems was negative for nausea, vomiting, diarrhea, shortness of breath (SOB), pain while taking a breath, cough, chest pain (CP), and skin lesions. She also denied any muscular pain, muscular weakness, or joint pain. The patient also denied any vaginal ulcerations or vaginal discharge. She did, however, admit to fevers and chills, and stated that the highest recorded temperature at home was 39.2°C. Vital signs upon presentation were as follows: temperature 38.1°C; blood pressure 108/73 millimeters of mercury; pulse 108 beats per minute; respiratory rate 16 breaths per minute, and oxygen saturation 99% on room air.

Physical examination revealed an African-American female sitting comfortably on the stretcher in no apparent respiratory distress. The patient appeared non-toxic, and conversation revealed no evidence of hoarse or muffled speech. A focused head, eyes, ears, nose, and throat exam revealed no oropharyngeal masses or uvular deviation, and no submental induration or tenderness were appreciated on examination. A bluish discoloration was appreciated on the tongue, but the patient admitted to using lozenges just prior to examination.

**Image 1:**
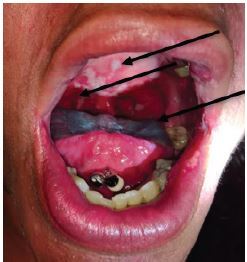
Raised corrugated plaques that could not be scraped off on exam, located on the oropharynx, soft palate, hard palate, and the side of her tongue.

**Image 2:**
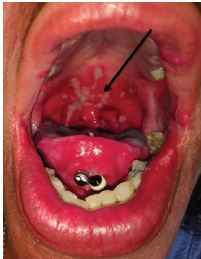
White, confluent, fluffy, hyperkeratotic lesions in a patient presenting with complaint of sore throat.

A focused examination of her oropharynx demonstrated mildly dry mucous membranes with confluent plaques and a white, patchy, ulcerative appearance of the uvula, tonsils, tonsillar pillars, hard palate, anterior one-third of the soft palate, and the side of her tongue (Images 1 and 2).

Given the patient’s pyrexia and symptomology, rapid streptococcal antigen and a mononucleosis spot (Monospot) test were obtained. The results for the Monospot test and rapid group A strep antigen screen were negative. KOH testing was performed on the posterior pharyngeal lesion for a possible fungal etiology and was found to be negative. Given the negative test results and appearance of the lesion, the patient was interviewed again. We specifically inquired about high-risk sexual behavior, IVDU, and using immunosuppressive medication, all of which the patient denied. She was then offered basic lab work consisting of a complete blood count (CBC) with differential and complete metabolic profile (CMP) (Table 1).

Based upon those results, a diagnosis was made in the ED, the patient was started on medications, and further laboratory work was ordered to confirm the diagnosis.

## CASE DISCUSSION

This 45-year-old African-American patient presented with a history of increasing sore throat over the course of three days, which was not relieved with OTC medications. The patient’s odynophagia led to decreased oral intake, which was accompanied by fevers and chills over those same three days. Causes for pharyngitis can be broadly placed into two categories: infectious and non-infectious causes.

### Non-Infectious

I initially considered non-infectious causes; however, as this was a clinicopathological case, I wanted to expand my differential diagnosis, and I felt those could be quickly excluded given her history and presentation (H&P) and the fact that the patient had a fever. A drug-induced presentation such as dental decay secondary to methamphetamine use was quickly excluded, as this mostly involves the teeth. I briefly considered ingestion of a foreign body, caustic or alkaline burns, and thermal burns, but this was quickly ruled out as this was not consistent with her H&P and by the fact that she had white plaques on examination. Stevens-Johnson syndrome and fixed drug reactions do have oral involvement, but can be quickly ruled out as there were no skin rashes on this patient.

I next turned my attention to autoimmune disorders, which can have oral involvement, and I considered serositis, myositis, and Sweet’s syndrome, but I quickly dismissed these diagnoses as the patient did not have systemic symptoms seen with these disorders. Specifically, she had denied the following: a) SOB, CP, and abdominal pain (serositis); b) muscular pain or weakness (polymyositis, dermatomyositis); and c) skin rash (dermatomyositis, Sweet’s syndrome).

The final causes I considered in the non-infectious category were neoplastic causes. Leukemia can present with thrush, but I quickly ruled this out, as this patient had a normal total white blood cell count, no systemic symptoms, and the scrapings of the oropharyngeal lesions had yielded a negative KOH preparation. The patient did admit to a history of smoking and, therefore, I strongly considered oropharyngeal cancer. While this diagnosis remained high on my differential diagnosis, I felt that the precipitous onset of symptoms in addition to the fever made this diagnosis less likely than an infectious cause and, therefore, I turned my attention to infectious causes.

### Infectious Causes

The most common causes for pharyngitis are of viral etiology, including infections due to rhinovirus, adenovirus, coxsackie, influenza, and Epstein-Barr virus (EBV). Considering the patient’s age and the extensive involvement of the lesions (Images 1 and 2), a simple viral infection was less likely. However, an infection with EBV causing mononucleosis should remain on the differential, keeping in mind that the heterophile test (Monospot test) can take over one week until it turns positive and in this case the patient’s symptoms have been present for only three days. However, mononucleosis often presents with monocytosis, which was not the case with this patient (Table 1), making mononucleosis a less likely diagnosis.

Bacterial causes of pharyngitis are very common, with the most common pathogen being *Streptococcus pyogenes*, aka group A beta-hemolytic streptococcal infection (GABHS), which can ultimately lead to rheumatic fever. However, considering the patient’s age, the negative rapid group A strep antigen screen, and the extensive involvement of the lesions, especially the hard palate and tongue, make a bacterial etiology unlikely. Further, the Centor Score on this patient was found to be only 2, giving the patient a low risk of 11–17% of GABHS.

Sore throat and odynophagia can also be a presentation of bacterial infection leading to a peritonsillar abscess, infection of the floor of the mouth such as Ludwig’s angina, or infection of the retropharyngeal or submandibular spaces. On physical examination, the patient did not have uvular deviation, tongue elevation, or signs of airway compromise, such as stridor, drooling, inability to tolerate secretions, muffled voice, or hypoxia, making these diagnoses less likely. These physical findings, in addition to the fact that the patient was up to date on her immunizations, made diagnoses such as epiglottitis, bacterial tracheitis, and diphtheria also less likely.

Another rare presentation to consider in this patient would be Lemierre’s syndrome, which is also known as infectious thrombophlebitis of the internal jugular vein caused by *Fusobacterium necrophorum*. In general, this disease is associated with lethargy, swollen lymph nodes, and a prolonged disease course leading to sepsis. This patient did not have any lymphadenopathy and while tachycardic with a mild fever, did not appear septic. Since this was a clinicopathology case, there was likely nothing common about it, and therefore the zebras had to be considered. It is unclear in this case whether recent orogenital contact exposure had occurred; therefore, I added gonococcal and herpes simplex virus (HSV) pharyngitis to the differential diagnosis.

At this point in time, I had almost ruled out the majority of my working differential diagnosis while a possibility for mononucleosis, oropharyngeal cancer, and possible gonococcal or HSV pharyngitis persisted, which led me to question what I was missing and if there were any further clues from this patient. The patient is a smoker, which increased the risk of oral or systemic cancers, but she denied IVD and alcohol use. She also denied risky sexual behavior and any current prescription medications or being immunosuppressed, thus making many of the above “less common causes” less likely.

In addition, while the patient denied using any prescription medications, I was wondering if there was a component of medication non-compliance that could point us in the right direction. Upon further exploration, I learned the patient had a history of bipolar disorder, depression, hypertension, GERD, and had no primary care physician, meaning she had no regular screenings or check-ups. These together call into question the reliability of the history of present illness (HPI), and whether she might have a higher risk of any type of infectious, immunosuppression, precancerous, or cancerous process. However, since I was unable to further derive much information from her HPI, I next focused on the laboratory results. The patient was found to have a normal absolute WBC cell count. Interestingly, the differential of the CBC results revealed lymphopenia with a lymphocyte percentage of 7.6%, which raised concern for immunosuppression given that the normal percent of lymphocyte count for an adult female CBC should fall in the range of 20–51% (Table 1). Further, while the normal absolute lymphocyte cell count in a healthy adult should be approximately 1.2–3.4 X 10^9^/L, the absolute lymphocyte count result in this case was 0.3 X 10^9^/L (Table 1). Furthermore, her low platelet counts of 117 × 10^9^/L (normal: 130–400 × 10^9^/L) sparked my interest. It is well known that thrombocytopenia is very common in patients with human immunodeficiency virus (HIV) and acquired immune deficiency syndrome (AIDS) and is often associated with disease progression.^1^

In conclusion, after pulling together the HPI, social history, patient’s fever, and physical exam findings, along with the finding of lymphopenia and thrombocytopenia, I was convinced that these were consistent with HIV/AIDS defining illness, likely HIV oral lesions (HIV-OL), specifically oral hairy leukoplakia (OHL).

## CASE OUTCOME

The patient was clinically diagnosed with OHL, and discharged with a prescription for high-dose acyclovir. The patient was informed that we suspected immunosuppression to be the cause of her lesions and further laboratory workup was ordered and sent for serological and immunological analysis. The earlier suspicion of HIV infection was confirmed a few days later, when infectious disease (ID) serologies detected HIV-1 RNA with a HIV-1 viral load totaling 192,000 copies/mL. Further, the patient was found to have reactive hepatitis-C antibodies (Table 2). The patient was also tested for syphilis, chlamydia, gonorrhea, and hepatitis A and B, all of which were found to be negative (Table 2).

The patient was contacted immediately and was referred to the local ID physician for further evaluation and treatment. She was started on antiretroviral therapy (ART). Further follow-up showed that the patient’s symptoms resolved after she was started on STRIBILD (elvitegravir/cobicistat/emtricitabine/tenofovir) and that she continued to follow up with the ID physician. Five months after the patient was started on STRIBILD, her absolute CD4 count had improved from her initial count of 192 cells/mm^3^ to 1029 cells/mm^3^. Her HIV-1 viral load had declined at that point in time to 131,000 copies/mL.

## RESIDENT DISCUSSION

Chief complaint of pharyngitis, or sore throat, represents one of the most common reasons for ED visits and accounts for a total of 2.6 million ED visits annually.^2^ The patient’s complaint is often manifested as a constellation of symptoms consisting of throat pain exacerbated by swallowing, which may or may not radiate to the ears and neck, prompting patients to present to the ED for evaluation. Various subtypes of pharyngitis are more prevalent among different patient populations. Swelling, erythema with or without exudate, and inflammation involving the soft tissues of the oropharynx are the causes for the clinical symptoms of pharyngitis, but any involvement of the anatomic structures in the hypopharynx or nasopharynx can also lead to a sore throat.^3^

Acute pharyngitis has a wide range of pathological etiologies, thus emergency physicians should consider a broad differential when encountering a patient with pharyngitis, especially if the H&P does not fit the presenting clinical picture. While some physicians choose to empirically treat pharyngitis with antibiotics, other emergency physicians use a variety of scoring systems to differentiate between viral, fungal, and bacterial etiology. A common decision tool used in the ED is the Centor Score decision tool to guide the diagnosis and treatment of GABHS. Most patients presenting to the ED with a sore throat will be diagnosed with uncomplicated infectious pharyngitis with a benign disease course. However, there is a small subset of patients who either present with an atypical presentation of pharyngitis or have a suspicious past medical history, which must prompt the practitioner to expand his or her differential diagnosis and diagnostic approach. One such subset is comprised of immunocompromised patients who can present with a variety of oral lesions. Unfortunately, in developed countries, and in the era of ART, physicians often do not consider differential diagnoses for oral lesions where undiagnosed HIV infection could be the underlying cause for such lesions.

Worldwide, infection with HIV is the most common cause leading to an immunocompromised physical state in patients and it presents a major global health issue. The prevalence of HIV-infected people worldwide in 2018 was estimated to be 37.9 million with an incidence rate of 1.7 million.^4^ In the United States, the prevalence is estimated to be 1.1 million with an incidence rate of ~40,000 people.^5^ In 2018, only 23.3 million infected individuals had access to ART worldwide, and the death toll secondary to AIDS-related illnesses was estimated to be 770,000 people.^4^ Further, it is estimated that 21% of all people worldwide and 14% of people in the US living with HIV do not know their HIV status and do not receive any treatment.^4^

The World Health Organization published a classification for HIV-OL, which has been the mainstay of defining lesions that are commonly observed in patients with HIV. The classification system separates HIV-OL into three categories: 1) oral lesions strongly associated with HIV-infection; 2) lesions less commonly associated with HIV infection; and 3) lesions directly observed in HIV infection.^6^ Oral candidiasis (OC) and OHL are the two most prevalent HIV-OL lesions that are observed in the group of oral lesions strongly associated with HIV infection.^6^ This was validated in a study that assessed oral lesions in ART naïve patients and found that OC followed by OHL have the highest incidence of HIV-OL.^7^ It is known that certain HIV-OL observed in patients with diagnosed or undiagnosed HIV have a positive correlation predicting the progression to AIDS.^8^–^10^

OHL is a clinical presentation that is found almost exclusively in untreated patients with advanced HIV. Further, OHL is the result of an acute EBV infection or reactivation of a latent EBV infection and results from an impaired oral mucosal cell-mediated immunity.^11^,^12^ This malfunction of oral mucosal immunity is believed to be multifactorial. It has been reported that the HIV virus indirectly impairs nitric oxide and lysozymes production and IgA synthesis and secretion by the oral mucosa that normally function as bactericidal, fungicidal, and viricidal agents leading to OHL.^12^ Most recently, it has been suggested that HIV impairs the oral mucosal barrier in addition to suppressing CD4+ T cells by also suppressing the Th17 T-cells, which secrete IL-17 and IL-23 cytokines, which further weakens the oral mucosal immunity and predisposes it to opportunistic infections.^12^,^13^

Grossly, OHL appears as white, patchy, raised, corrugated tissue overgrowth present on the lateral surfaces of the tongue, hard palate, and oropharynx, and the projected material present on the surface contributes to the “hairy” appearance.^14^,^15^ The visualized lesions are more common on the tongue, and are often more prevalent in males as compared to females.^16^ Pathognomonically, these lesions are unable to be scraped away during examination, which is often useful in separating it from OC.^14^,^15^ Microscopically and histologically, OHL is characterized by a benign stratified squamous epithelial hyperplasia with characteristic pallor.^14^,^15^ While OHL is considered a “benign” oral manifestation of HIV infection, it is of the utmost importance to diagnose this lesion due to the fact that it can represent the first physical sign of significant immunosuppression in HIV-infected patients. Clinical appearance alone is often sufficient for diagnosis. However, if confirmation is required, liquid-based cytology with in situ hybridization for EBV can be used for diagnosis and is a simple, noninvasive tool that is equivalent to traditional punch biopsy of the lesion.^17^ OHL usually resolves after the patient is started on ART therapy, but some clinicians have advocated starting a patient on acyclovir or valacyclovir upon diagnosis OHL, especially if the patient is symptomatic.^10^,^15^

HIV viral load has been shown to have a positive correlation to the development of oral lesions, as a viral load of 30,000 copies/mm^3^ exhibited oral lesions related with HIV, independent of a patient’s total CD4 cell count.^18^ Commonly, the median CD4+ count when OHL is first visualized by practitioners ranges between 235–468 cell/mm^3^.^2^,^9^ Oral lesions are generally an early sign of HIV infection, but could also be used to predict the clinical manifestations and progression of HIV/AIDS infections in patients.^4^ Literature demonstrates an inverse relationship between the development of OHL and declining CD4 + counts and it has been shown that HIV infection with OHL often progresses to AIDS with CD4 counts of less than 200 cell/mm^3^.^5^,^10^ Further elucidating the inverse relationship between the presence of OHL and declining CD4+ counts, one study found that “the probability of developing AIDS at the time of HIV diagnosis with the presence of OHL when not receiving ART is 48% by 16 months and 83% at 31 months, respectively.”^13^,^18^

While some oral lesions observed with OHL have historically been identified as an “AIDS defining illness,” it should be noted that in recent years patients have presented with OHL and other oral lesions who were not infected with HIV.^15^ Patients with immunosuppression secondary to various etiologies, such as solid organ transplant on maintenance anti-rejection medications, chronic steroids use, or hematologic malignancies, have all presented clinically with OHL.^7^,^15^ This case report demonstrates that atypical pharyngeal lesions should prompt the treating physician to consider immunosuppression as a possible etiology. It is noteworthy to add that some emergency physicians have been hesitant in the past to perform HIV testing in the ED for various reasons including difficulty following up on results, inability to counsel patient on results, and lack of access to follow-up.^19^–^22^ The authors disagree with this notion and, as is demonstrated in this case, it is the duty of the emergency physician to order testing so that an appropriate diagnosis and treatment referral can be given to the patient.

In 2006 the CDC updated the HIV testing guidelines to include testing all patients who present to healthcare facilities with concern for possible infection, including the ED.^19^–^22^ It is recommended that counseling should be provided to the patient prior to HIV testing, and also after testing, if the results are in fact positive.^19^–^22^ A cross-sectional analysis using data from two large national healthcare surveys from the years 2009–2014 was performed and found that HIV testing in EDs was nationally low, despite the updated CDC recommendation in 2006.^20^ The conclusion reached by the authors is that testing for HIV should be performed in the ED, especially in patients who solely rely on ED visits for their healthcare.^20^ In conclusion, emergency physicians must be reminded that HIV-OL may present in the ED setting and physicians should consider serological testing while in the ED so that the appropriate follow-up and treatment for the patient can be initiated.

## FINAL DIAGNOSIS

Oral hairy leukoplakia, presenting as an AIDS defining illness. Reactive hepatitis C antibody, indicating current or prior hepatitis C infection.

## KEY TEACHING POINTS

- Pharyngitis is common in the emergency department (ED), accounting for 2.6 million visits annually, and is most often caused by viral, bacterial, and fungal pathogens.
- Atypical presentations of pharyngitis should prompt the ED provider to expand their differential diagnosis, especially if there are concerns for possible immunosuppression.
- Oral hairy leukoplakia (OHL) is the second most prevalent HIV defining oral lesion, after candida.
- OHL is typically a white, patchy area on oral mucosa that is unable to be scraped off, and may have pedunculated tissue, hence the “hairy” descriptor.
- OHL has historically been an “AIDS defining illness,” but can also been seen with solid organ transplant immunosuppression, chronic steroid use, or hematologic malignancies.

## Figures and Tables

**Table 1:** Complete blood count with differential and complete metabolic panel.

Complete blood count		Reference range
WBC	4.6	4.5–11.0 × 10^9^/ L
RBC	4.03 L	4.20–5.40 ×10^12^cell/ L
Hgb	14.4	12.0–16.0 g/L
Hct	40.0	37–47%
MCV	99.3	81–101 fL
MCH	35.8 H	26–34 pg
MCHC	36.0	32–36 g/L
RDW	11.7	11.5–14.5%
Plt Count	117 L	130–400 × 10^9^/L
Granulocyte	88.2 H	42.2–75.2 %
Lymphocytes	7.6 L	20.5–51.1 %
Monocytes	3.8	1.7–9.3 %
Eosinophils	0.1	0–5 %
Basophils	0.3	0–3 %
Absolute Granulocytes	4.0	1.4–6.5 × 10^9^/L
Absolute Lymphocytes	0.3	1.2–3.4 × 10^9^/L
Absolute Monocytes	0.17	0.0–0.82 × 10^9^/L
Absolute Eosinophils	0.0	0.0–0.52 × 10^9^/L
Absolute Basophils	0.01	0.0–0.2 × 10^9^/L
Complete metabolic panel		
Sodium	138	137–145 mmol/L
Potassium	3.7	3.5–5.2 mmol/L
Chloride	99	98–107 mmol/L
Carbon dioxide	30	22–30 mmol/L
Anion gap	9	3 and 10 mEq/L
BUN	11	7–17 mg/dL
Creatinine	0.71	0.52–1.04 mg/dL
BUN/CR ratio	15.5	10–20
Calculated osmolality	262 L	275–295 mOsm
Glucose	85	74–106 mg/dL
Calcium	8.5	8.4–10.2 mg/dL
T. Bilirubin	0.7	0.2–1.3 mg/dL
AST	24	14–36 U/L
ALT	18	9–52 U/L
Alkaline Phosphate	95	38–126 U/L
Albumin	3.3 L	3.5–5.5 g/dL

*WBC*, white blood cell count; *RBC*, red blood cell count; *HgB*, hemoglobin; *Hct*, hematocrit; *MCV*, mean corpuscular volume; *fL*, femtoliter; *MCH*, mean corpuscular hemoglobin; *pg*, picogram; *MCHC*, mean corpuscular hemoglobin concentration; *RDW*, red cell distribution width; *Plt*, platelet count; *BUN*, blood urea nitrogen, *CR*, creatinine; *mOsm*; milliosmole; *T*, total; *AST*, aspartate aminotransferase; *ALT*, alanine aminotransferase; *mmol*, mllimole; *mg*, mlligrams; *dL*, deciliter; *U*, units; *L*, liter; *g*, grams.

**Table 2:** Infectious disease serology and immunology of patient with suspected immunosuppression.

Test	Result	Reference range
Treponema pallidum Ab	Nonreactive	Nonreactive
*C. trachomatis* RNA	Not Detected	Not detected
Cryptococcus Ag Screen	Not Detected	Not detected
Hepatitis A IgM Ab	Nonreactive	Nonreactive
Hepatitis B Antigen	Nonreactive	Nonreactive
Hepatitis B Core IgM Ab	Nonreactive	Nonreactive
Hepatitis C Antibody	Reactive H	Nonreactive
HIV-1 RNA log copies/mL	5.28 H	1.30–7.00 log copies/mL*
HIV- RNA PCR copies/mL	192000 H	20–10,000 copies/mL*
HIV Genotype	Detected	Not detected
HIV-2 Antibody Conf	Negative	Negative
Infectious Mono Assay	Negative	Negative
*N. Gonorrhoeae* RNA	Not Detected	Not detected
Absolute CD4 Count	192 L	600–1,200 cell/mm^3^
HLA-B57.01	Negative	Negative

* Assay quantification result range.

*C*, chlamydia; *RNA*, ribonucleic acid; *H*, high; *L*, low; *HIV*, human immunodeficiency virus; PCR, polymerase chain reaction; *mono*, mononucleosis; *N*, Neisseria.
